# Dose-response study of propofol combined with two different doses of esketamine for laryngeal mask airway insertion in women undergoing hysteroscopy

**DOI:** 10.1016/j.heliyon.2024.e30511

**Published:** 2024-04-30

**Authors:** Yan-Jun Lin, Su-Li Chen, Xiang-Li Zheng, Shuang Yu, Liang-Yuan Lu

**Affiliations:** Department of Anesthesiology, Aerospace Center Hospital, Beijing, 100049, China

**Keywords:** Esketamine, Propofol, Median effective dose, Laryngeal mask airway, Dose-response relationship

## Abstract

**Objective:**

To prospectively determine the median effective dose (ED50) of propofol for inhibiting a response to laryngeal mask airway (LMA) insertion when combined with different doses of esketamine in female patients.

**Methods:**

A total of 58 female patients (aged 20–60 years, ASAⅠ–Ⅱ) scheduled for elective hysteroscopy were enrolled and randomly divided into 2 groups, one of which was administered 0.2 mg/kg of esketamine (K1 group, n = 28) and the other 0.3 mg/kg of esketamine (K2 group, n = 30). The 2 groups received the corresponding doses of esketamine intravenously, followed by an intravenous injection of propofol (injection time was 30 s). The initial dose of propofol was 2 mg/kg, and the dose ratio of propofol in the adjacent patients was 0.9. If a positive reaction occurred due to LMA insertion, the dose ratio in the next patient was increased by 1 gradient; if not, the dose ratio was decreased by 1 gradient. The ED50, 95 % effective dose (ED95) and 95 % confidence interval (CI) of propofol for inhibiting a response to LMA insertion in the 2 esketamine groups were calculated using probit analysis.

**Results:**

The ED50 of propofol for inhibiting a response to LMA insertion in female patients was 1.95 mg/kg (95 % CI, 1.82–2.08 mg/kg) in the K1 group and 1.60 mg/kg (95 % CI, 1.18–1.83 mg/kg) in the K2 group. The ED95 of propofol for inhibiting a response to LMA insertion in female patients was 2.22 mg/kg (95 % CI, 2.09–2.86 mg/kg) in the K1 group and 2.15 mg/kg (95 % CI, 1.88–3.09 mg/kg) in the K2 group.

**Conclusion:**

Propofol combined with 0.3 mg/kg of esketamine has low ED50 and ED95 effective doses for inhibiting an LMA insertion response in female patients undergoing hysteroscopy and surgery. There were no significant adverse effects, but the additional dose of propofol and airway pressure were significantly higher than those in the group administered 0.2 mg/kg of esketamine. Based on the results, we recommend the combination of propofol with 0.2 mg/kg esketamine for optimal conditions during LMA insertion in women undergoing hysteroscopy.

## 1 Introduction

The laryngeal mask airway (LMA) is a supraglottic airway device that was first introduced by Brain in 1983 [1]. Its placement does not require laryngoscopy, and it stimulates fewer airway reflexes and sympathetic responses from supraglottic placement compared with endotracheal intubation. However, because muscle relaxants are not generally used when inserting the LMA, adequate inhibition of the upper airway reflex is required. The choice of anaesthetic drugs and their dosages can affect the quality of anaesthesia provided by the LMA during surgery. Specifically, the selected anaesthetic technique influences how effectively the LMA maintains a patent airway, allows for adequate ventilation, and prevents intraoperative complications such as coughing, gagging, or patient movement. Additionally, the anaesthetic approach can impact the incidence of postoperative respiratory adverse events, including coughing, laryngospasm, airway obstruction, or desaturation. Inappropriate or inadequate suppression of airway reflexes may cause these issues, which, in turn, can lead to suboptimal LMA placement or even placement failure [2].

Propofol is a commonly used short-acting intravenous anaesthetic inducer that can effectively suppress the upper airway reflexes, relax the lower jaw and reduce the sensitivity of the upper airway, providing optimal LMA placement conditions; accordingly, it is widely used in the placement of LMA during the general anaesthesia induction period [3]. Although propofol is an effective hypnotic drug, it cannot provide effective analgesia [4]. However, propofol alone appears to have several dose-dependent adverse effects, such as respiratory depression, hypotension, hypoxaemia and injection pain. Using propofol as the sole anaesthetic inducer reduces the success rate of LMA placement [5], but co-induction agents, such as μ-receptor opioids, remifentanil, fentanyl and sufentanil can be combined with propofol to reduce any propofol-related adverse events in clinical practice, thereby improving the insertion conditions and LMA insertion success rate [[6], [7], [8], [9]].

Esketamine is a left-handed isomer of ketamine that can be administered intranasally. However, in this study, esketamine was administered intravenously at different doses. Because it has a higher affinity for N-methyl-D-aspartate (NMDA) receptors than the right-handed enantiomer (R)-ketamine, its anaesthetic effect is twice that of (R)-ketamine. As a non-competitive antagonist of the NMDA receptor, esketamine is more effective than competitive antagonists because it can bind to the receptor at a different site than the agonist, thereby preventing receptor activation regardless of the agonist concentration. This makes non-competitive antagonists more potent and effective at inhibiting the receptor's response over a wider range of agonist concentrations [10]. It has the dual effect of sedation and analgesia and can increase the sympathetic tone. In addition, the excitatory effect on the cardiovascular system can partially offset the inhibitory effect of propofol, making the heart rate and blood pressure more stable. Small doses can exert equivalent sedative and analgesic effects to ketamine and can maintain stable haemodynamics and a low incidence of adverse effects.

In preclinical research and clinical applications, esketamine is mostly used in the research and treatment of psychiatric disorders and depression [11]. However, there are currently no studies comparing the application of esketamine combined with propofol to LMA insertion.

The sequential test technique is a test design method. It does not specify the number of trials, but after each trial or series of trials, it determines whether to continue the trial according to a preset stop rule until the stop rule is met. A decision analysis is then performed. The advantage of this method lies in finding the optimal value of a factor as soon as possible with as few trials as possible [12]. The present study observed the effect of different doses of esketamine on a median effective dose (ED50) and a 95 % effective dose (ED95) combined with propofol for inhibiting the LMA placement response in female patients using the sequential test method.

## 2 Data and methods

### 2.1 Clinical data

A total of 58 patients undergoing gynaecological hysteroscopy and surgery with elective general anaesthesia in our hospital were included in this prospective study.

Inclusion criteria: 1) women undergoing gynaecological hysteroscopy and surgery resulting from any indication; 2) patients aged from 20 to 60 years old; 3) body mass index (BMI): 18–30 kg/m^2^; 4) American Society of Anesthesiologists physical status score: grade I–II [13]; 5) voluntary participation in the study. Exclusion criteria: 1) pregnant and lactating women; 2) patients with a history of propofol allergy, uncontrolled hypertension, hyperthyroidism, severe cardiopulmonary diseases, airway stenosis, mental illness or gastroesophageal reflux disease; 3) those who have recently taken or are taking psychotropic drugs or analgesic drugs; 4) patients who were allergic to or addicted to esketamine. Based on the esketamine dose, and using computer-generated random selection, patients were randomly divided into a group that was administered 0.2 mg/kg of esketamine (K1) and a second group that was administered esketamine of 0.3 mg/kg (K2). Allocation concealment was ensured using thick sealed envelopes, one containing the details of group A (K1, 0.2 mg/kg) and another that of group B (K2, 0.3 mg/kg).

This study was approved by the Ethics Committee of the Aerospace Center Hospital (Beijing Aerospace Medicine Review 2022 No.068). The patients and family signed informed consent forms for inclusion in the research.

### 2.2 Methods

None of the patients had been administered preoperative medication. After the patient arrived at the operating theatre (OT), their upper-extremity venous access was established through intravenous catheter insertion in the pre-operative area. Once in the operating room, Ringer's lactate solution was infused through the intravenous line. Routine monitoring consisted of an electrocardiogram, non-invasive blood pressure, pulse oxygen saturation (SpO_2_), capnography and bispectral index (BIS) monitoring [14]. An oxygen mask was provided. Subsequently, the sealed envelope with the patient allocation was opened. In both groups, esketamine was diluted into a 10-ml syringe by an anaesthesiologist who had not participated in the case collection. The drugs were then given to the OT technician. Patients in the K1 group received 0.2 mg/kg of esketamine (2ml:50 mg; Jiangsu Hengrui Pharmaceutical Co., Ltd., H20193336) intravenously over 30 s, and those in the K2 group received 0.3 mg/kg of esketamine intravenously over 30 s. Ten seconds after esketamine administration, propofol was injected; after waiting for another 120 s and following the disappearance of the eyelash reflex, a laryngeal mask (LMA® Supreme™) was inserted (number 3 for patients weighing 30–50 kg, and number 4 for patients weighing 50–70 kg). Standard LMA insertion techniques were used, including jaw thrust, chin lift and rotating the LMA 180° if needed to optimise positioning. To avoid any technical errors, all the LMA placements in this study were performed by the same anaesthesiologist.

The test was performed using the sequential method. The initial dose of propofol was 2 mg/kg, after which an LMA was easily inserted. For the next patient, the propofol dose was increased or decreased by 10 % of the previous patient's dose. If there was a positive reaction to the LMA insertion, the gradient was increased for the next patient; otherwise, the gradient was decreased, and the study was terminated after 7 crossover inflexion points.

After successful LMA placement, a ventilator was used to control breathing, with the tidal volume set to 8 ml/kg and a respiratory rate of 10–12 times/min. Anaesthesia was maintained using a continuous intravenous infusion of 4–8 mg/kg/h propofol and a BIS of 40–60. After surgery, the LMA was removed following spontaneous breathing and the patient recovering consciousness. The patient was sent to the anaesthesia recovery room and, once fully awake, to the ward.

### 2.3 Observation indicators

① A record of the patient's response during LMA insertion was taken and included the following (Table 1): body movement or no body movement, where ‘body movement’ was defined as a cough, breath-holding or laryngospasm when the LMA was inserted or the laryngeal cuff inflated, and the patient had difficulty opening their mouth and experienced conscious movement throughout the body. ‘No body movement’ was defined as the absence of these reactions when the LMA was inserted, or the laryngeal cuff inflated. ② Peak airway pressure was observed and recorded within 5 min after a successful LMA insertion. The peak airway pressure values were recorded using a ventilator pressure monitor. ③ The overall condition of LMA insertion was reviewed as follows: the mouth opening ≥3 cm was considered complete, whereas the mouth opening <3 cm was considered incomplete. The LMA insertion conditions were as follows: 1 = complete relaxation, 2 = mild resistance, 3 = resistance but can open the mouth, 4 = resistance and the need to further increase the propofol dose. Here, scores 1 and 2 were considered successful LMA insertion and scores 3 and 4 failure [14]. If the patient experienced body movement when the LMA was inserted, intravenous propofol of 0.5–1 mg/kg was added, and insertion was halted temporarily, and then resumed. ④ The following values were recorded: mean arterial pressure (MAP), heart rate (HR), SpO_2_ rate before anaesthesia (T0),1 min before LMA insertion (T1), 1 min after LMA insertion (T2) and 2 min after LMA insertion(T3). Tidal volume and airway pressure were controlled after LMA insertion [15]. ⑤ Additional propofol doses and adverse reactions in patients who tested positive during the induction process (e.g. hypotension, bradycardia and apnoea). If the patient developed bradycardia (<45 beats/min), 0.3–0.5 mg of intravenous atropine was given, and 1–2 mg of intravenous dopamine was administered for hypotension (<20 % of basal blood pressure) (Table 1).

**Table 1:** Response scoring scale of LMA insertion.

Evaluation indicator	Excellent	Good	Poor
Swallow	No	Mild	Obvious
Nausea and Vomiting	No	<2 times	>2 times
Body movement	No	Yes, small	Yes, large
Laryngismus	No	Part	Complete

### 2.4 Statistical analysis

Line statistical data were analysed using the SPSS 22.0 statistical software. Normally distributed measurement data are expressed as mean ± standard deviation, and comparisons between different time points were analysed using repeated measures analysis of variance. A pre-test pilot study was conducted with a smaller sample size (n = 10 in each group) to estimate the expected effect size and variability. Based on the pilot study results, a sample size of 26 patients per group was determined as sufficient to detect a statistically significant difference with a power of 0.8 and an alpha level of 0.05. The ED50 and ED95 of esketamine and the corresponding 95 % CIs were calculated using probit probability. Sequential plots and dose-effect plots were plotted using Microsoft Excel software. A result denoting P < 0.05 was considered a statistically significant difference.

## 3 Results

### 3.1 General information

**Fig. 1:**
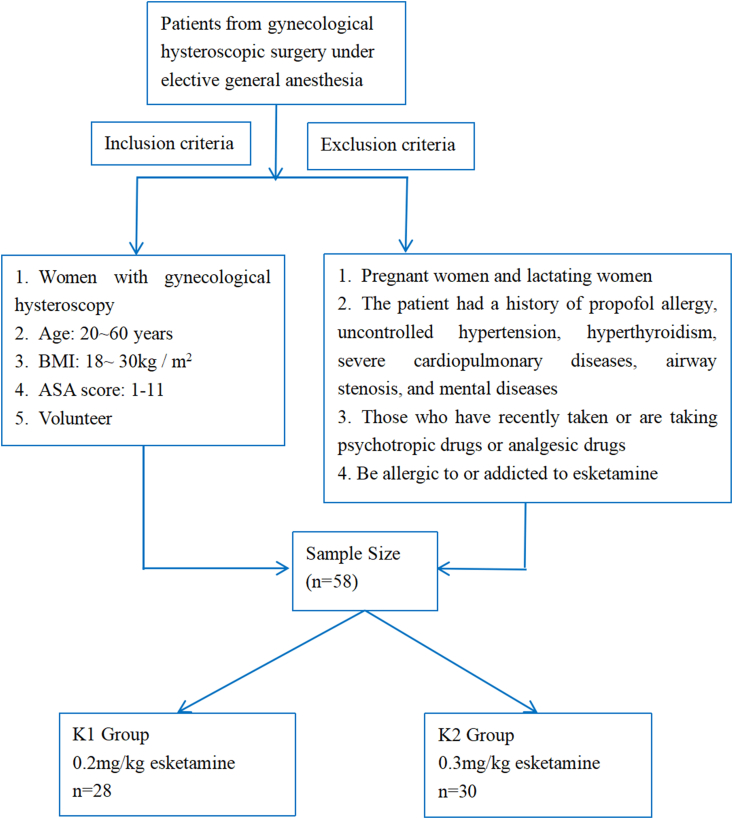
CONSORT flow diagram.

A total of 58 patients were recruited for this study, with 28 in the K1 group (aged 45.4 ± 8.7 years; BMI, 23.4 ± 3.6 kg/m^2^) and 30 in the K2 group (aged 44.9 ± 8.1 years; BMI, 23.4 ± 2.2 kg/m^2^). A CONSORT flow diagram is presented in Fig. 1. No significant differences were observed in the general condition of the patients in the 2 groups.

The intraoperative BIS monitoring of both groups was 50–60, and an LMA was successfully inserted for all patients. No endotracheal intubation was performed. Compared with T0, MAP and HR decreased at T1, T2 and T3 without any statistical differences between the 2 groups (both P > 0.05), and there was no difference in SpO_2_ (P > 0.05) (Table 2).

**Table 2:** Comparison of hemodynamic changes at different time points in both groups ($\bar{x}$ ±s).

	MAP (mmHg)				HR (beats/min)			
	T0	T1	T2	T3	T0	T1	T2	T3
K1 group	90.8 ± 10.9	89.5 ± 11.8	85.3 ± 9.2	82.7 ± 10.6	73.4 ± 9.6	72.8 ± 10.3	68.4 ± 10.3	68.6 ± 8.5
K2 group	91.9 ± 12.3	88.8 ± 10.6	82.5 ± 9.7	79.6 ± 9.8	74.0 ± 10.2	73.5 ± 10.8	71.3 ± 10.6	68.3 ± 8.8

Table 3 shows the peak airway pressure within 5 min after LMA insertion in the 2 groups. The peak airway pressure was significantly higher in the K2 (0.3 mg/kg esketamine) compared to the K1 (0.2 mg/kg esketamine) group, with a statistically significant difference (P < 0.001).

**Table 3:** Peak airway pressure (cmH_2_O).

Group	Mean ± SD
K1 group	13.5 ± 2.1
K2 group	16.6 ± 2.8

### 3.2 Median effective dose and 95 % effective dose

**Fig. 2:**
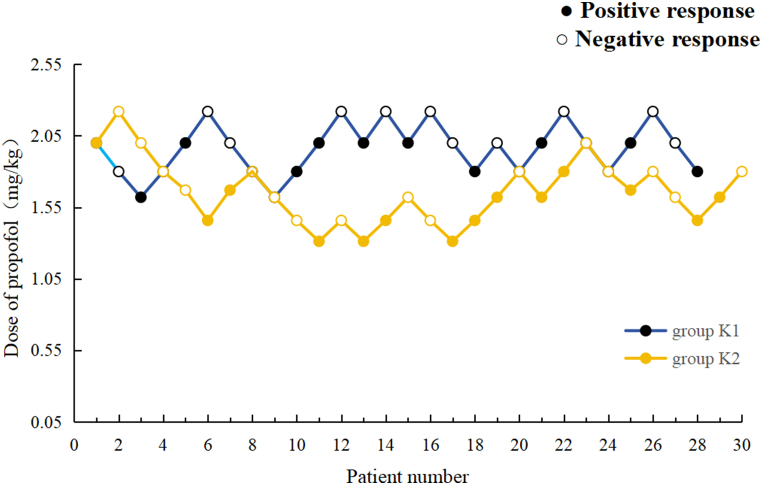
Sequential diagram of propofol inhibiting response of LMA insertion in both groups.

**Fig. 3:**
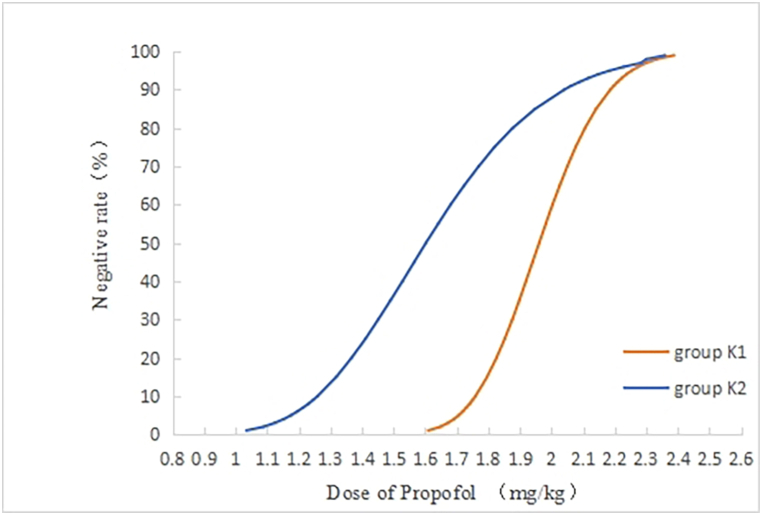
Dose-effect plot of propofol inhibiting response of LMA insertion in both groups.

During the operation, 14 patients in the K1 group had a positive response and 14 had a negative response (Fig. 2). In patients with a positive response, the additional dose of propofol was 42.3 ± 7.6 mg, tidal volume was 472.5 ± 40.9 ml and airway pressure was 13.5 ± 2.1 mmHg after LMA placement. The ED50 of propofol for inhibiting a response to the LMA placement in the K1 group was 1.95 mg/kg (95 % CI, 1.82–2.07 mg/kg) and the ED95 was 2.22 mg/kg (95 % CI, 2.08–2.66 mg/kg). In the K2 group, 14 patients had a positive response and 16 had a negative response (Fig. 2). In patients exhibiting a positive response, the additional dose of propofol administered after the LMA placement was 56.9 ± 8.9 mg. The tidal volume recorded was 472.5 ± 42.0 ml, and the airway pressure was 16.6 ± 2.8 mmHg. The ED50 of propofol for suppressing the response to LMA insertion in the K2 group was determined as 1.60 mg/kg (95 % CI, 1.18–1.83 mg/kg), and the ED95 was 2.15 mg/kg (95 % CI, 1.88–2.48 mg/kg). Dose-response curves depicting the relationship between propofol doses and their efficacy in inhibiting responses to the LMA insertion in female patients across both groups are presented in Fig. 3. The additional doses of propofol and airway pressure were significantly higher in the K2 than those in K1 group.

### 3.3 Adverse reactions

No corresponding circulatory and psychiatric adverse reactions were observed in patients. Those with a positive response successfully received an LMA after additional propofol, and no adverse reactions occurred during or after surgery.

## 4 Discussion

This study found that compared with the group that was administered 0.2 mg/kg of esketamine, the ED50 (1.60 and 1.95 mg/kg) and ED95 (2.15 and 2.22 mg/kg) doses of propofol for inhibiting a response to LMA insertion in the group that was administered 0.3 mg/kg of esketamine were low, and the LMAs were all successfully inserted. However, the additional doses of propofol (56.9 and 42.3 mg) and airway pressure (16.6 and 13.3 mmHg) in this group were significantly higher. The preliminary experimental data of this study also revealed that the ED50 of esketamine, combined with 2 mg/kg of propofol for inhibiting the LMA placement response, was 0.168 mg/kg (95 % CI, 0.157–0.181 mg/kg) and the ED95 was 0.192 mg/kg (95 % CI, 0.179–0.256 mg/kg), suggesting that the haemodynamics were stable and the success rate of LMA insertion was high in cases that received 2.0 mg/kg of propofol combined with 0.2 mg/kg of esketamine.

Propofol is commonly used to induce laryngeal mask ventilation in clinical surgery of short duration. It is the most commonly used clinical intravenous anaesthetic drug and aids muscle relaxation and the inhibition of throat reflex. It has thus become the most effective drug for use in LMA placement under non-muscle relaxation conditions [16]. However, when administered alone, a large dose of propofol is required to achieve optimal LMA placement conditions via the drug's inhibitory effects on circulation and respiration. In hysteroscopic surgery, opioids are avoided as much as possible. A recent meta-analysis and review of 23 randomised controlled trials demonstrated that postoperative pain scores were consistent between opioid and non-opioid treatment groups; however, in comparison, non-opioid anaesthesia significantly reduced the incidence of postoperative nausea and vomiting (PONV) [17]. The 0.2 mg/kg esketamine dose makes this low-opioid anaesthetic regimen the preferred choice for patients at high risk of PONV. Therefore, propofol is often combined with opioid or non-steroidal analgesic drugs for LMA placement to reduce the adverse effects of propofol and analgesic drugs [18].

Esketamine is an isomer of ketamine. In the hypnotic and anaesthetic states, small doses of esketamine do not affect the respiratory inhibitory effect of propofol. As a non-competitive NMDA receptor antagonist, it has a stronger affinity to the NMDA receptor, acts twice as fast as ketamine, and has the characteristics of rapid metabolism, few adverse reactions and rapid recovery [19]. In addition, it has the dual effects of sedation and analgesia and can increase the sympathetic tone, with the excitatory effect on the cardiovascular system partially offsetting the inhibitory effect of propofol, making the HR and blood pressure more stable. Small doses can exert equivalent sedative and analgesic effects to ketamine [20], maintaining stable haemodynamics and a low incidence of adverse effects [21]. Recently, Zheng et al. designed a four-arm randomised controlled trial in children undergoing diagnostic upper gastrointestinal endoscopy to investigate propofol doses combined with different doses of esketamine for inducing an appropriate depth of anaesthesia in 50 % of patients [22]. The results showed that the total doses of propofol in groups that were administered 0.5 and 1 mg/kg of esketamine were statistically lower compared with groups that were administered 0 and 0.25 mg/kg of esketamine (P < 0.01). Song et al. measured the ED50 of propofol for inhibiting the excitatory circulation of low-dose ketamine in 21 patients under general anaesthesia using the sequential method [23]. The ED50 of propofol was 0.56 mg/kg, and the 95 % CI was 0.54–0.62. The results showed that propofol could effectively inhibit the sympathetic excitatory effect of low-dose ketamine. Blood pressure and SpO_2_ were stable and respiratory depression was slight when the 2 different drugs were combined.

In this study, no patients experienced significant haemodynamic changes after drug administration or within 3 min of the LMA insertion, demonstrating that a small dose of esketamine, combined with propofol, could stabilise drug-induced haemodynamic fluctuations during the LMA insertion. Additionally, none of the patients experienced severe hypertension or psychiatric/neurological adverse effects, indicating that the compound application of low-dose esketamine with propofol is safe.

This study has some limitations, such as a small sample size and including only female patients aged 20–60 years. Further research is needed to investigate the response of LMA insertion in male patients and patients in other age ranges using the same methods.

## 5 Conclusion

Propofol combined with 0.3 mg/kg of esketamine resulted in lower ED50 and ED95 values for inhibiting the response to an LMA insertion in female patients undergoing hysteroscopy and surgerythan those in the group administered 0.2 mg/kg of esketamine. These patients remained haemodynamically stable, had a high placement successful rate and experienced no significant adverse effects. But the additional dose of propofol and airway pressure was significantly higher than those in the 0.2mg/kg esketamine group. We recommend using propofol in combination with 0.2 mg/kg esketamine to achieve optimal insertion conditions and reduce potential airway complications during LMA-guided anaesthesia for hysteroscopic procedures in female patients.

## Ethics approval and consent to participate

This study was reviewed and approved by the Ethics Committee of Aerospace Center Hospital, with the approval number: JHYL2022No.068.

All participants provided informed consent to participate in the study.

## Funding

Not applicable.

This research did not receive any funding support.

## Data availability

Has data associated with your study been deposited into a publicly available repository?

No, No data was used for the research described in the article.

## Consent for publication

The manuscript is not submitted for publication or consideration elsewhere.

## CRediT authorship contribution statement

**Yan-Jun Lin:** Writing – original draft, Resources, Methodology, Formal analysis, Data curation, Conceptualization. **Su-Li Chen:** Writing – original draft, Resources, Methodology, Investigation, Formal analysis, Data curation. **Xiang-Li Zheng:** Writing – review & editing, Resources, Methodology, Investigation, Formal analysis, Data curation. **Shuang Yu:** Writing – review & editing, Resources, Methodology, Formal analysis. **Liang-Yuan Lu:** Writing – review & editing, Supervision, Software, Methodology, Investigation, Formal analysis, Data curation.

## Declaration of competing interest

The authors declare that they have no known competing financial interests or personal relationships that could have appeared to influence the work reported in this paper.
